# ASO Author Reflections: Increasing National Performance on Complete Tumor Resection in Patients with Gastric Cancer by Awareness of Risk Factors and Network Organization for Gastric Cancer Surgery

**DOI:** 10.1245/s10434-019-07887-7

**Published:** 2019-10-03

**Authors:** Leonie R. van der Werf, Bas P. L. Wijnhoven

**Affiliations:** Department of Surgery, Erasmus University Medical Centre Rotterdam, Rotterdam, The Netherlands

## Past

For 9–13% of patients with gastric cancer who underwent gastrectomy with curative intent, the resection margins are tumor-positive.^1^ It is important to gain insight about which patients are at risk for an incomplete tumor resection. Therefore, our study was designed to evaluate the factors associated with incomplete tumor removal.^2^

Previous studies also showed that higher hospital volume is associated with improved surgical quality and outcome. This has resulted in centralization of gastric cancer surgery. In 2016, 9 of 22 hospitals were low-volume hospitals for gastrectomy.^3^ The present study also evaluated whether an incomplete tumor resection is associated with hospital volume.

## Present

This Dutch cohort study showed that patients with advanced gastric cancers (i.e., involving the entire stomach, advanced TNM-stage and diffuse-type gastric cancer) are at risk for incomplete tumor removal. These risk factors may lead to a change in surgical strategy. For example, an extra wide tumor resection margin may be the target or intraoperative frozen-section analysis may prevent incomplete tumor removal. In addition, when it is anticipated that the proximal margin at the esophagus or the duodenum is at risk, it may be indicated to refer patients to hospitals where esophageal and/or hepatobiliary surgery is performed.

Another important finding in this study was that low annual hospital volume (< 20 resections per year) was associated with a higher risk for incomplete tumor removal compared with middle- and high-volume hospitals. This finding may point toward the need for further centralization of gastric cancer surgery or to discuss all patients in a multidisciplinary team of gastric cancer experts.^4^

## Future

Unfortunately, due to the retrospective design of this study, the influence of treatment-related factors, such as neoadjuvant chemotherapy and the surgical approach on the completeness of the resection, could not be evaluated. A strength of this study was the nationwide coverage of the database allowing assessment of national performance. However, the use of a national database also has limitations. Because the data for this study were retrieved from the database of the Dutch Upper Gastrointestinal Cancer Audit, some surgical details were lacking. This national audit includes outcomes that are used to evaluate and compare performances between hospitals. Because it is time-consuming to register surgical details, these are not registered in the audit. To further evaluate these surgical details, we have recently started another study. In this study, specific data regarding surgical details and treatment details will be collected.
